# Trehalose-6-Phosphate Phosphatase I (TPPI) Regulates Floral Transition, Nitrogen Responses, and Photosynthetic Performance in Arabidopsis

**DOI:** 10.3390/plants15172559

**Published:** 2026-08-23

**Authors:** Behzad Heidari, Dugassa Nemie-Feyissa, Amr R. A. Kataya, Peter Ruoff, Cathrine Lillo, Lutz Andreas Eichacker

**Affiliations:** 1 IKBM, Department of Chemistry, Bioscience and Environmental Engineering, University of Stavanger, 4036 Stavanger, Norway; 2 Department of Agroecology, Faculty of Technical Sciences, Aarhus University, 4200 Slagelse, Denmark

**Keywords:** trehalose-6-phosphate, TPPI, *Arabidopsis thaliana*, flowering time, nitrogen assimilation, nitrate reductase, photosynthesis, carbon signalling, Dual-PAM, T6P signalling

## Abstract

Trehalose-6-phosphate (T6P) is a key signalling metabolite that integrates carbon availability with development and stress responses in plants. T6P levels are controlled by trehalose phosphate synthase (TPS) and trehalose-6-phosphate phosphatase (TPP) enzymes; however, while TPS enzymes have been studied extensively, the physiological functions of individual TPPs remain incompletely understood. Here, we investigated the role of TPPI in Arabidopsis using loss-of-function *tppi* mutants, a complemented line (*tppi*+35S::*TPPI*), and *TPPI*-overexpressing (*TPPI-OEX*) plants. The *tppi* mutant exhibited delayed flowering accompanied by reduced expression of *CO*, *FT*, and *SPL3*, while complementation restored wild-type (WT) flowering time. *TPPI-OEX* plants displayed an intermediate flowering phenotype with moderate reductions in *CO* and *FT* expression. Under nitrogen starvation, *tppi* plants showed enhanced anthocyanin accumulation, altered nitrate reductase regulation, characterised by lower total enzyme activity but a higher activation state, and enhanced expression of nitrate assimilation and uptake genes (*NIA1*, *NIA2*, *NRT1.1*, and *NRT2.1*). TPPI deficiency also altered photosynthetic performance, with enhanced photosystem I (PSI) acceptor-side limitation, increased non-photochemical quenching (NPQ), and a tendency toward reduced photosystem II (PSII) electron transport, indicating altered photosynthetic electron transport and energy dissipation. Taken together, these results indicate that TPPI contributes to the regulation of flowering time, nitrogen responses, and photosynthetic performance, suggesting broader effects of TPPI on plant developmental and physiological processes.

## 1. Introduction

Trehalose-6-phosphate (T6P) is a pivotal signalling molecule integrating metabolic and developmental processes in Arabidopsis. T6P is synthesised from UDP-glucose and glucose-6-phosphate by trehalose phosphate synthase (TPS) enzymes and subsequently dephosphorylated to trehalose by trehalose-6-phosphate phosphatases (TPPs) [1]. Arabidopsis possesses 11 *TPS* genes (Class I: *AtTPS1*–4; Class II: *AtTPS5*–11) and 10 *TPP* genes (*AtTPPA–AtTPPJ*), suggesting that T6P homeostasis is regulated through a complex multigene network [2,3].

The precursors of T6P are derived from sucrose metabolism [4]. T6P levels closely track sucrose availability, and sucrose rapidly induces a substantial increase in T6P [5,6], enabling T6P to signal sucrose status and coordinate carbon utilisation, photosynthetic capacity, carbon allocation, and growth [7,8,9,10]. Thus, appropriate regulation of T6P levels is important for coordinating carbon availability with plant growth and development.

T6P signalling has been linked to several physiological processes relevant to plant productivity. Recent studies on wheat have demonstrated that chemical manipulation of T6P signalling can increase yield by enhancing both source activity, including CO_2_ fixation and electron flow, and sink processes such as starch, amino acid, and protein synthesis [11]. The photosynthetic apparatus is one important target of this regulation. Plants overexpressing bacterial *otsA* (*TPS*) exhibit enhanced photosynthetic capacity, whereas *otsB* (*TPP*) overexpression has the opposite effect, indicating that T6P contributes to the regulation of photosynthesis and leaf development [12,13,14]. Similar effects were reported in Arabidopsis plants overexpressing *BnaC02.TPS8* from *Brassica napus* [15].

Beyond its effects on photosynthesis, T6P regulates starch metabolism, including both starch synthesis and turnover. T6P regulates starch biosynthesis through ADP-glucose pyrophosphorylase (AGPase) and controls its breakdown during energy demand [16]. In this way, T6P helps coordinate starch turnover with sucrose availability. In addition to controlling carbon flux from starch to sucrose synthesis, elevated T6P levels promote phosphoenolpyruvate carboxylase (PEPC) and nitrate reductase (NR) activation, thereby linking carbon status with nitrogen assimilation and metabolic adjustment [17,18].

T6P-mediated diversion of photoassimilates away from sucrose biosynthesis can be explained partly by the inhibition of sucrose non-fermenting 1-related protein kinase 1 (SnRK1) by T6P. SnRK1 is primarily activated by low-energy conditions, which are often associated with stress. Upon activation, SnRK1 promotes metabolic adaptation by inhibiting anabolic processes and stimulating catabolic pathways [19,20]. SnRK1 is known to phosphorylate and inactivate SUCROSE PHOSPHATE SYNTHASE (SPS), NR and PEPC enzymes [17,18]. In Arabidopsis, overexpression of *otsA* and *otsB* genes alters responses to low-nitrogen conditions. Overexpression of *otsA* increases sensitivity to low-nitrogen conditions, leading to increased anthocyanin accumulation. Anthocyanin accumulation is a common plant response to nitrate depletion and contributes to tolerance of nitrogen limitation. In contrast, *otsB*-overexpressing plants resembled those with *SnRK1* (*KIN10*) overexpression, showing reduced anthocyanin and delayed senescence [12]. More recently, overexpression of *BnaC02.TPS8* in Arabidopsis was also shown to increase sensitivity to low N stress and high sucrose levels, accompanied by increased anthocyanin accumulation [21].

In addition to its metabolic roles, T6P levels in leaves serve as a gauge of resource availability, influencing developmental pathways including the regulation of flowering time. T6P coordinates flowering by integrating photoperiodic and carbohydrate-dependent signals. T6P regulates flowering through the photoperiodic pathway via the *CONSTANS (CO)–FLOWERING LOCUS T (FT)* regulatory network. Elevated T6P levels promote *FT* expression and subsequent floral initiation, while low T6P levels repress *FT*, delaying flowering. T6P also regulates flowering through the age pathway by repressing *miR156*, thereby promoting *SQUAMOSA PROMOTER BINDING PROTEIN-LIKE (SPL)* gene expression and the transition to flowering [22]. In addition to TPS enzymes, *TPP* genes also contribute to flowering-time regulation in plants. Overexpression of *otsB*, several *AtTPPs*, and *JcTPPJ* from *Jatropha curcas* has been reported to delay flowering in Arabidopsis [3,23,24].

Although the biochemical functions of TPS and TPP enzymes in T6P metabolism are well established, the physiological roles of individual TPP isoforms remain incompletely understood. Importantly, disruption of individual TPP genes does not necessarily result in T6P accumulation. Analyses of Arabidopsis single *tpp* mutants have revealed variable effects on T6P abundance, with T6P levels increasing, decreasing, or remaining largely unchanged depending on the affected isoform [3]. Notably, *tppi* mutants show reduced rather than increased T6P levels [3,25], despite the loss of a T6P phosphatase. This unexpected response distinguishes *tppi* from the simple expectation for loss of TPP activity and points to isoform-dependent regulation of T6P homeostasis. Among the Arabidopsis TPP family members, TPPI is predicted to localise to multiple subcellular compartments, suggesting that it may perform functions distinct from those of other TPP isoforms. In this study, we analysed the function of TPPI in Arabidopsis using *tppi* mutants and a *TPPI*-overexpressing (*TPPI-OEX*) line. Our results show that altered *TPPI* expression affects flowering time, nitrogen responses, and photosynthetic performance, supporting a broader role for TPPI in plant developmental and physiological processes.

## 2. Results

### 2.1. Flowering Time

**Figure 1:**
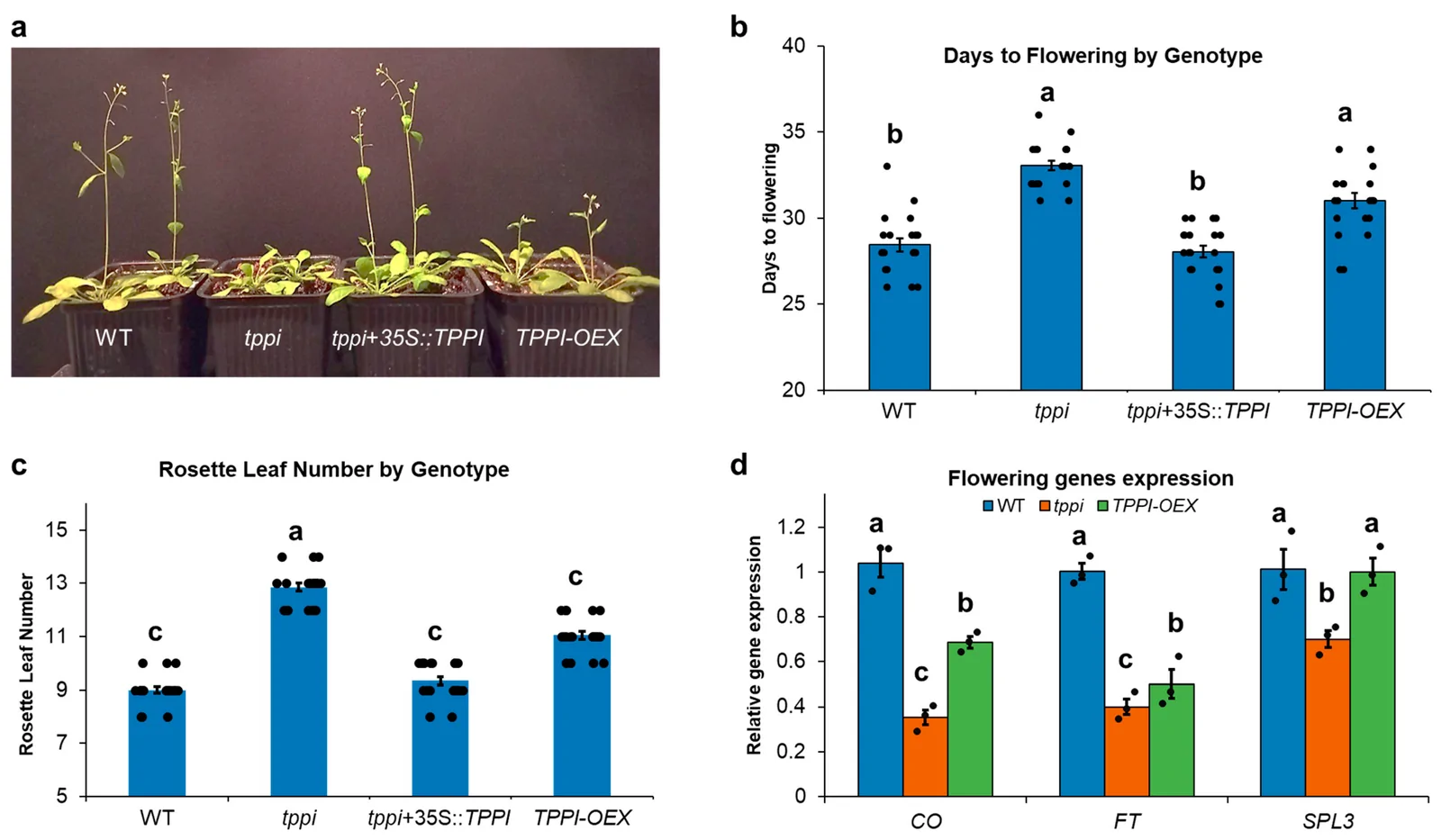
TPPI regulates flowering time in Arabidopsis. (**a**) Representative plants of WT, *tppi*, the complemented line (*tppi*+35S::*TPPI*) and *TPPI-OEX* under long-day conditions (16 h light/8 h dark). (**b**) Days to flowering. (**c**) Rosette leaf number at bolting. For (**b**,**c**), values are means ± SE (*n* = 20 plants per genotype), with individual data points shown. Different letters indicate significant differences (one-way ANOVA followed by Tukey’s HSD test, *p* < 0.05). (**d**) Relative expression of *CO*, *FT* and *SPL3*. Expression was normalised to WT (=1). Values are means ± SE (*n* = 3 biological replicates). Individual data points are shown. Different letters indicate significant differences within each gene (one-way ANOVA followed by Tukey’s HSD test, *p* < 0.05).

We assessed flowering time under long-day conditions (16 h light/8 h dark) in four genotypes: wild type (WT), *tppi* mutant, complemented line (*tppi*+35S::*TPPI*), and *TPPI-OEX* plants. The *tppi* mutant flowered significantly later than WT plants, while the complemented line flowered at a time comparable to that of WT, supporting the conclusion that the delayed-flowering phenotype results from the loss of TPPI function. *TPPI-OEX* plants also exhibited delayed flowering, although the phenotype was less pronounced than in the *tppi* mutant. Thus, the flowering time of *TPPI-OEX* plants was intermediate between WT and *tppi* plants (Figure 1a,b). Consistent with the delayed flowering phenotype, *tppi* plants also produced significantly more rosette leaves before bolting than WT plants, whereas the complemented line resembled WT and *TPPI-OEX* plants showed an intermediate phenotype (Figure 1c).

To identify the flowering pathways affected, we analysed the expression of key marker genes associated with flowering regulation, including *CO* from the photoperiodic pathway and *SPL3* from the age pathway, together with the floral integrator, *FT*. The *tppi* mutant showed significantly reduced expression of *CO*, *FT*, and *SPL3*, with *CO* and *FT* exhibiting stronger reductions than *SPL3*. In *TPPI-OEX* plants, *SPL3* expression remained close to WT levels, whereas *CO* and *FT* expression were moderately reduced compared with WT but remained higher than in *tppi* mutants (Figure 1d). Additional flowering-related genes, including *FLC*, *MYB33*, *SOC1*/*AGL20*, and *MIR156A*, were also examined. No statistically significant differences in the expression of these genes were observed between genotypes, although *FLC* expression tended to be slightly higher in *tppi* plants.

### 2.2. TPPI Regulates Nitrogen Assimilation

**Figure 2:**
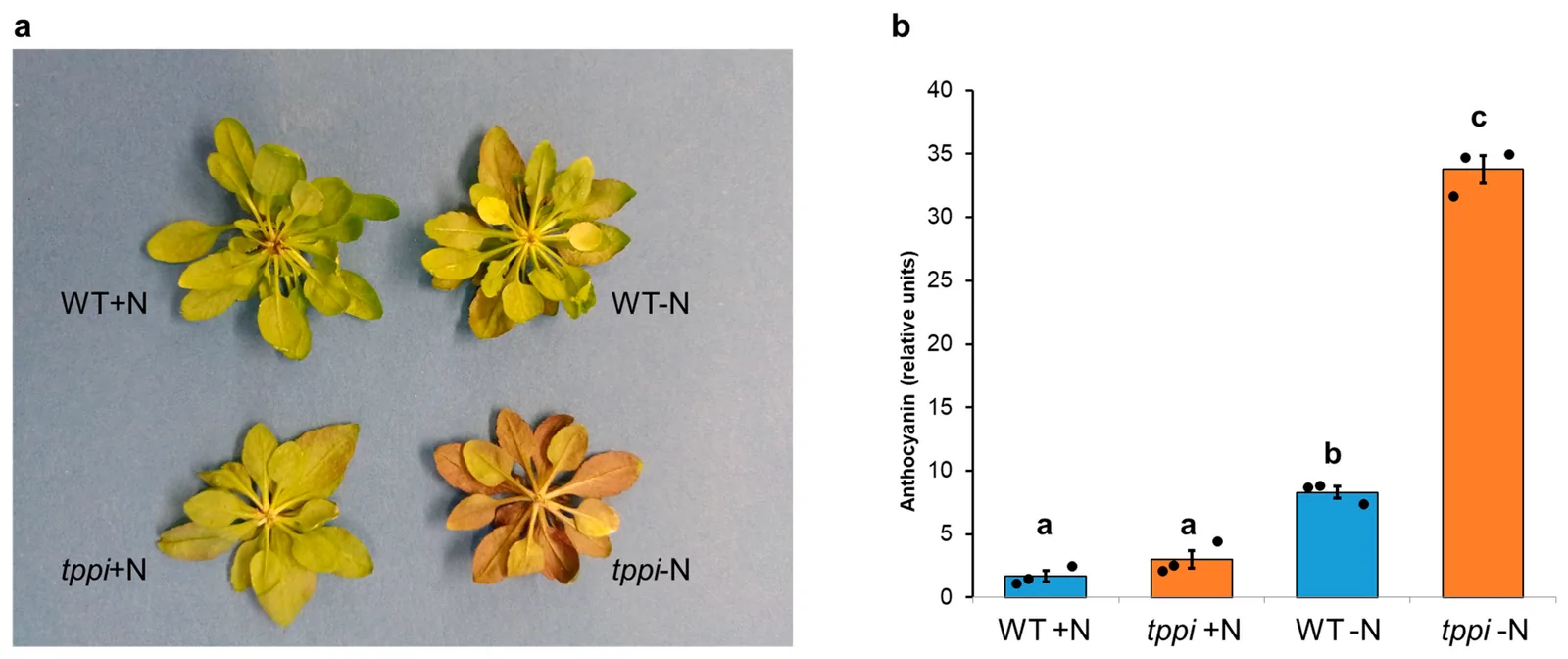
TPPI affects responses to nitrogen starvation. (**a**) Representative rosettes after transfer to +N or −N nutrient solution, showing stronger anthocyanin accumulation in *tppi* under −N. (**b**) Anthocyanin content (A_530_ − A_657_). Values are means ± SE (*n* = 3 biological replicates). Individual data points are shown. Data were analysed by two-way ANOVA with genotype and nitrogen treatment as factors. A significant genotype × nitrogen-treatment interaction was detected (*p* < 0.0001). Different letters indicate significant differences between groups based on Tukey’s HSD multiple-comparison test (*p* < 0.05).

WT and *tppi* plants were grown for three weeks on complete nutrient solution and then transferred to either +N (control) or −N conditions. Anthocyanin accumulation is a common plant response to nitrate depletion. Under nitrogen depletion conditions, *tppi* mutant plants accumulated more anthocyanin than WT plants (Figure 2a). To quantify this response, we measured anthocyanin content in rosette leaves under +N and −N conditions. Anthocyanin levels were low in both WT and *tppi* under +N, but increased under −N in both backgrounds, with the highest accumulation observed in *tppi* plants (Figure 2b). The quantitative measurements were consistent with the visual phenotype in Figure 2a and indicated that nitrogen starvation elicited a stronger anthocyanin response in the *tppi* background. The complemented and *TPPI-OEX* lines were also examined under nitrogen-replete and nitrogen-starvation conditions. Neither line differed significantly from WT in anthocyanin accumulation under the conditions tested (Supplementary Figure S2a).

**Figure 3:**
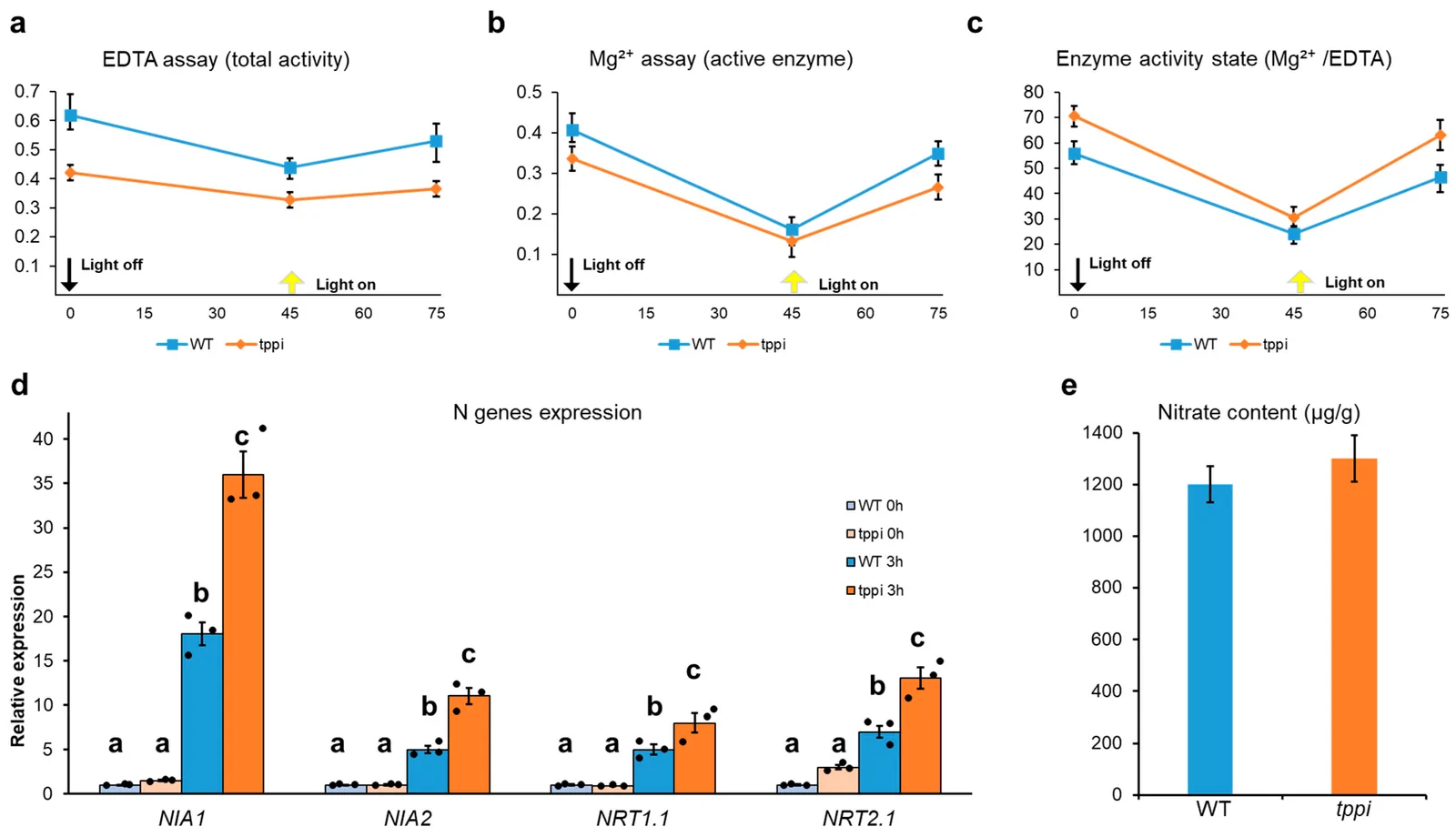
TPPI influences nitrate reductase activity and nitrate-responsive gene expression. (**a**) Total nitrate reductase (NR) activity (EDTA assay). (**b**) Actual NR activity (Mg^2+^ assay). (**c**) NR activation state (Mg^2+^/EDTA × 100). (**d**) Relative expression of *NIA1*, *NIA2*, *NRT1.1*, and *NRT2.1* at 0 h and 3 h after nitrate resupply following nitrogen starvation. Expression was normalised to WT at 0 h. Values are means ± SE (*n* = 3 biological replicates). Individual data points are shown. For each gene, data were analysed by two-way ANOVA with genotype and time as factors, followed by Tukey’s HSD multiple-comparison test. Significant genotype × time interactions were detected for *NIA1* (*p* = 0.000317), *NIA2* (*p* = 0.000344), *NRT1.1* (*p* = 0.0361), and *NRT2.1* (*p* = 0.0209). Different letters indicate significant differences between groups (*p* < 0.05). (**e**) Leaf nitrate content. Values are means ± SE (*n* = 3 biological replicates per genotype). Individual data points are shown. No significant difference was detected between genotypes (two-tailed unpaired Student’s *t*-test, *p* = 0.68).

Given the greater anthocyanin accumulation observed in *tppi* mutants under nitrogen starvation, we examined NR activity to assess potential alterations in nitrogen assimilation associated with the *tppi* mutation. The NR activation state represents the percentage of non-phosphorylated (active) NR relative to total NR activity, which includes both non-phosphorylated and phosphorylated forms [26]. The phosphorylated form of NR is specifically inhibited in the presence of Mg^2+^ during the assay, so only the non-phosphorylated NR remains active. However, when EDTA is added to the assay, it chelates Mg^2+^ and disrupts 14-3-3-mediated inhibition, thereby activating the phosphorylated form of NR as well. WT plants showed higher total NR activity (EDTA assay) (Figure 3a) and, correspondingly, higher actual NR activity (Mg^2+^ assay) (Figure 3b). Notably, the proportion of active NR (Mg^2+^/EDTA) was higher in *tppi* mutants compared with WT (Figure 3c). NR activity was also measured in *TPPI-OEX* plants, but no significant difference from WT was detected under the conditions tested.

To determine whether the reduced total NR activity in *tppi* mutants could be explained by altered transcription of the genes encoding NR, we first examined the expression of *NIA1* and *NIA2* under nitrogen-replete (+N) conditions. Under these conditions, no significant differences in *NIA1* or *NIA2* expression were detected between WT and *tppi* plants, indicating that the reduced NR activity is not attributable to lower basal expression of these genes (Supplementary Figure S2b).

Because basal *NIA1* and *NIA2* expression was unaffected, we next investigated whether TPPI influences the transcriptional response of nitrate assimilation and transport genes to changes in nitrogen availability. Plants were therefore subjected to nitrogen starvation, and the expression of *NIA1*, *NIA2*, *NRT1.1*, and *NRT2.1* was analysed immediately before nitrate resupply (0 h) and 3 h after nitrate addition. Transcript levels were similarly low in both genotypes at 0 h. Following nitrate resupply, however, all four genes were induced, with significantly stronger induction in *tppi* plants than in WT, particularly for *NIA1*, whereas *NIA2*, *NRT1.1*, and *NRT2.1* showed more moderate increases (Figure 3d).

To determine whether this enhanced transcriptional response was accompanied by increased nitrate accumulation, we measured leaf nitrate content. Despite the stronger induction of nitrate assimilation and transport genes, nitrate content did not differ significantly between *tppi* mutants and WT plants (Figure 3e).

### 2.3. TPPI Regulates Photosynthesis

Previous work demonstrated that *tppi* seedlings display enhanced hypocotyl elongation on sucrose-containing medium [27]. This finding suggests that sucrose alleviates the inhibition of hypocotyl elongation in *tppi* mutants and points to an important role for sucrose metabolism during early development.

Mutants of *tppi* also show altered levels of several central sugars. Previous studies demonstrated that *tppi* mutants contain lower amounts of starch and sucrose compared with WT plants. Notably, despite the TPP mutation, which might be expected to increase T6P levels, *tppi* mutants instead contain lower T6P levels than WT plants, similar to observations reported for *tppa*, *tppf*, and *tppb* mutants [3]. Because photosynthesis is the primary source of carbohydrates in plants, we further investigated whether TPPI affects photosynthetic performance.

**Figure 4:**
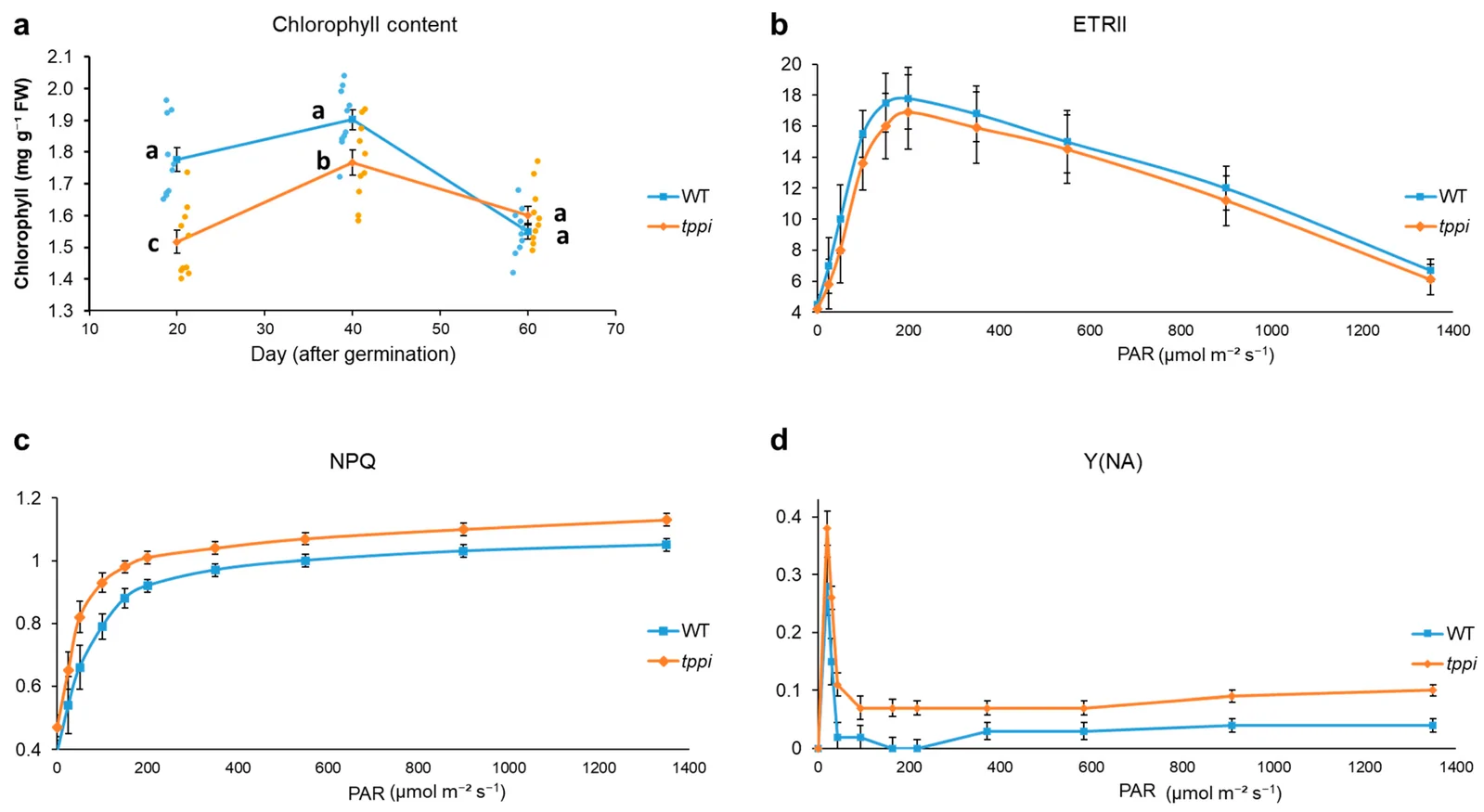
TPPI regulates photosynthetic performance. (**a**) Chlorophyll content in WT and *tppi* plants during development under short-day conditions. Values are means ± SE (*n* = 10 biological replicates per genotype at each developmental time point) with individual data points shown. Data were analysed by two-way ANOVA with genotype and developmental time as factors. A significant genotype × time interaction was detected (*p* = 0.000108). Different letters indicate significant differences between groups based on Tukey’s HSD multiple-comparison test (*p* < 0.05). (**b**–**d**) Light-response curves of (**b**) ETR(II), (**c**) NPQ, and (**d**) Y(NA). Values are means ± SE (*n* = 10 independent leaves per genotype). Light-response curves were analysed by two-way repeated-measures ANOVA, with genotype as the between-subject factor and PAR as the within-subject factor. Significant overall genotype effects were detected for NPQ and Y(NA) (*p* < 0.0001), but not for ETR(II) (*p* = 0.109).

We found that the *tppi* mutant exhibits a distinct pattern of chlorophyll accumulation over time. During the early weeks of growth under short-day conditions, *tppi* plants showed lower chlorophyll content than WT plants. As the plants matured, however, the chlorophyll content in *tppi* gradually became slightly higher than that in WT plants (Figure 4a). This pale-leaf phenotype was not observed in the complemented or *TPPI-OEX* lines (Supplementary Figure S3a).

To further investigate photosynthetic performance, we used a Dual-PAM-100 system to assess PSII and PSI function. Light-response curves showed that the PSII electron transport rate [ETR(II)] tended to be lower in *tppi* plants than in WT across the light-response curve, although the overall response pattern was similar between genotypes (Figure 4b). In contrast, *tppi* mutants displayed consistently higher NPQ across the light-response curve (Figure 4c).

The induction curves also showed altered qL behaviour, consistent with changes in PSII excitation pressure (Supplementary Figure S3b). For PSI, the quantum yield of acceptor-side limitation [Y(NA)] was higher in *tppi* plants than in WT, indicating greater restriction of electron transfer to downstream acceptors (Figure 4d). Together, these findings indicate altered photosynthetic energy dissipation and photosystem regulation in the *tppi* mutant (Figure 4a–d). Dual-PAM measurements were also performed in the complemented and *TPPI-OEX* lines, but neither showed significant differences from WT under the conditions tested.

## 3. Discussion

TPPI is one of ten Arabidopsis trehalose-6-phosphate phosphatase (TPP) isoforms, but its specific physiological role remains largely unexplored. Here, we investigated the physiological function of TPPI in Arabidopsis using a *tppi* loss-of-function mutant, a complemented line, and *TPPI-OEX* plants. Our results show that TPPI contributes to the regulation of flowering time, nitrogen responses, and photosynthetic performance, revealing effects of TPPI across multiple developmental and physiological processes.

The *tppi* mutant exhibited a significant delay in flowering under long-day conditions, while the complemented line fully restored WT flowering time. The restoration of flowering time in the complemented line supports the involvement of TPPI in this phenotype rather than secondary effects of the mutation (Figure 1a–c). Interestingly, *TPPI-OEX* plants also exhibited delayed flowering, with a mean flowering time intermediate between WT and the *tppi* mutant. Consistent with the phenotypic data, *tppi* mutant showed strongly reduced expression of the key flowering regulators *CO*, *FT*, and *SPL3*, whereas *TPPI-OEX* plants exhibited moderately reduced *CO* and *FT* transcript levels but near wild-type *SPL3* expression (Figure 1d). Together, these findings indicate that TPPI contributes to flowering time control through both the photoperiodic (*CO*–*FT*) and age-dependent (*SPL3*) pathways, consistent with previous studies linking T6P signalling to the regulation of floral transition [22].

The flowering phenotype of *TPPI-OEX* plants is notable because it indicates that both reduced and elevated *TPPI* expression can be associated with delayed flowering. Similar delayed-flowering phenotypes have been reported following the overexpression of several *TPP* genes, including *otsB*, *AtTPPs*, and *JcTPPJ* in Arabidopsis [3,23,24]. Contrary to expectations, the *tppi* mutant accumulates lower T6P levels than WT despite the loss of a T6P phosphatase, while trehalose is slightly elevated [3,25]. In *TPPI-OEX* plants, trehalose levels are strongly increased, whereas T6P levels are reduced [25]. The similar flowering phenotype observed in both *tppi* mutant and *TPPI-OEX* plants may be associated with their common reduction in T6P levels despite opposite changes in *TPPI* expression.

The counterintuitive decrease in T6P in the *tppi* mutant indicates that T6P abundance cannot be predicted solely from the activity of a single TPP isoform. Indeed, analyses of different *tpp* single mutants have shown highly variable effects on T6P levels (increased, decreased, or unchanged), pointing to compensatory mechanisms and regulatory interactions within the TPP family [3]. Recent studies with higher-order *tpp* mutants further support the view that individual TPP isoforms operate within a complex, interconnected network rather than functioning in isolation [28].

These observations raise the possibility that TPPI influences the activity of other TPP isoforms, directly or indirectly, thereby affecting overall T6P homeostasis. Previous studies have demonstrated redundant and non-redundant functions among Arabidopsis TPP isoforms and described regulatory features relevant to T6P metabolism [29,30]. A model which illustrates how mutual inhibitions (or activations) among TPP isoforms could generate non-intuitive changes in T6P levels is presented in the Supplementary Material “Modeling TPP crosstalk”. The kinetic formulations used in the model follow established enzyme-kinetic principles [31]. Analytical solutions and numerical simulations demonstrating the behaviour of the model under different assumptions are provided in Supplementary Figures S5–S8 and by the Supplementary Python programs FigureS7.py and FigureS8.py. As the model has not been experimentally validated, these simulations are intended to illustrate possible network behaviours rather than to provide evidence that such interactions occur in vivo. The resulting hypotheses therefore remain to be tested experimentally.

Beyond its developmental role, TPPI also contributes to metabolic adaptation under nutrient stress. The rapid anthocyanin accumulation in *tppi* mutants under low nitrogen conditions indicates an altered response to nitrogen limitation. To further examine this nitrogen-related phenotype, we assessed NR activity, a key enzymatic step in nitrate assimilation. Despite lower total NR activity, as measured in the EDTA assay, *tppi* mutants exhibited a higher proportion of active NR (Mg^2+^/EDTA ratio), which may reflect a compensatory regulatory response affecting NR activity. Elevated expression of *NIA1*, *NIA2*, *NRT1.1*, and *NRT2.1* in *tppi* plants further supports altered regulation of nitrate-responsive pathways following nitrate resupply in the absence of TPPI. However, nitrate content in *tppi* mutants was not significantly higher than in WT plants, indicating that the altered transcriptional response was not accompanied by increased nitrate accumulation. The combination of increased *NRT1.1* and *NRT2.1* expression, enhanced *NIA1* and *NIA2* induction, and an elevated NR activation state suggests that TPPI deficiency alters regulatory responses associated with nitrogen assimilation rather than bulk nitrate accumulation. This interpretation is consistent with the stronger anthocyanin response observed under nitrogen starvation, indicating altered physiological adaptation to nitrogen limitation.

This pattern contrasts with previous findings in which increased T6P levels resulting from *otsA* or *TPS8* overexpression promoted anthocyanin accumulation under low nitrogen conditions [12,21], suggesting that TPS- and TPP-mediated perturbations of T6P metabolism may affect nitrogen responses differently. Previous studies have also linked T6P signalling to the regulation of NR and related metabolic processes [18], providing a possible context for the altered nitrogen responses observed in *tppi*.

Because nitrogen assimilation and photosynthesis are closely interconnected in plant metabolism, we next examined how TPPI affects photosynthetic performance. The *tppi* mutants displayed a distinct chlorophyll accumulation pattern, with lower chlorophyll content during early growth stages under short-day conditions but higher levels than WT as plants matured. This pattern is consistent with the visibly paler phenotype of young *tppi* plants (Supplementary Figure S3a) and suggests that TPPI may influence chlorophyll accumulation during plant development. Previous studies have linked T6P signalling to chloroplast function and photosynthetic capacity, suggesting a potential connection with the altered chlorophyll accumulation observed in *tppi* [32].

Dual-PAM analysis revealed distinct changes in photosystem responses in *tppi* mutants. Higher Y(NA) values indicated increased PSI acceptor-side limitation, suggesting greater restriction of electron transfer beyond PSI. ETR(II) tended to be lower in *tppi* than in WT across the light-response curve, whereas NPQ was consistently higher, indicating enhanced photoprotective energy dissipation. Together, these findings indicate altered coordination of PSI and PSII responses in *tppi* plants. Induction kinetics further support these differences (Supplementary Figure S3b–d). In addition to these photosynthetic changes, lower starch and sucrose levels previously reported in *tppi* mutants [6], together with enhanced hypocotyl elongation on sucrose-containing medium [27], suggest altered carbohydrate metabolism and responses to sugar availability in the mutant. These findings align with earlier studies showing that manipulation of the trehalose pathway via *otsA* and *otsB* alters photosynthetic capacity, in part through effects on Rubisco expression and activity [14]. Overall, our results support the conclusion that TPPI deficiency is associated with altered chlorophyll accumulation, photosynthetic energy dissipation, and PSI acceptor-side limitation.

The changes observed in flowering time, nitrogen assimilation, and photosynthetic performance in *tppi* mutants suggest that TPPI may have broader effects on plant development and metabolism. These diverse effects may partly relate to the relationship between TPPI activity and T6P homeostasis. T6P acts as a central signal of sucrose availability and is a well-established inhibitor of SnRK1 activity. Previously reported alterations in T6P levels in *tppi* mutants [3,25] raise the possibility that changes in T6P–SnRK1 signalling could contribute to some of the observed phenotypes. SnRK1 activity has been shown to regulate gene expression through several transcriptional regulators, including bZIP transcription factors, and to influence protein synthesis and broader metabolic processes [33]. Thus, the T6P–SnRK1 pathway provides a possible framework for interpreting how changes in TPPI activity could influence multiple regulatory processes at both transcriptional and post-transcriptional levels.

However, this framework alone may not fully account for all of the observations reported here, and a direct mechanistic link remains to be established. One indication of such additional regulation is that *NIA1* and *NIA2* transcript levels were not reduced in *tppi* mutants, whereas total NR activity was lower, indicating that the effect on NR cannot be explained simply by reduced basal transcription and may involve additional metabolic or post-translational regulation. Similarly, the increased anthocyanin accumulation in *tppi* despite the previously reported reduction in T6P levels [3,25] contrasts with the association between elevated T6P and increased anthocyanin accumulation reported previously [13,22], suggesting that the nitrogen-starvation response is unlikely to be determined by T6P abundance alone. Together, these observations point to additional regulatory layers that may operate alongside T6P–SnRK1 signalling. Additional regulatory possibilities are suggested by studies of other TPP proteins. For example, the maize TPP RAMOSA3 (RA3) interacts with TPS proteins in a protein complex involved in the regulation of meristem development, indicating that TPP proteins can participate in regulatory protein interactions in addition to their enzymatic role [34]. Although comparable interactions have not been demonstrated for Arabidopsis TPPI, this finding raises the possibility that individual TPP isoforms may have interaction-dependent or isoform-specific functions that are not solely reflected by changes in bulk T6P abundance.

Overall, our results indicate that TPPI contributes to several aspects of plant development and physiology, including flowering time, nitrogen responses, and photosynthetic performance. The flowering response is particularly notable because both loss of *TPPI* and *TPPI* overexpression delayed flowering, although the effect was stronger in the *tppi* mutant, whereas complementation restored flowering to WT levels. This pattern appears distinct from that reported for other TPP genes. For example, loss or downregulation of Arabidopsis *TPPJ* results in earlier flowering, whereas overexpression of the Jatropha *TPPJ* orthologue in Arabidopsis has been reported to delay flowering [24,35]. Moreover, T6P levels in the *tppj* mutant were comparable to WT [3], whereas reduced T6P levels have been reported in both *tppi* and *TPPI-OEX* plants [3,25]. These contrasting responses suggest that individual TPP isoforms may influence flowering through distinct regulatory relationships with T6P homeostasis rather than through a simple relationship between TPP abundance and flowering time. Beyond flowering, the altered nitrogen responses and photosynthetic performance of the *tppi* mutant further indicate that TPPI function extends across multiple physiological processes. Together, these observations support an isoform-specific role for TPPI within the broader TPP network, although the regulatory basis of these effects remains to be established. Further comparative studies of individual TPP isoforms will be important for determining how TPP-specific regulation contributes to T6P homeostasis and downstream developmental and metabolic responses.

## 4. Materials and Methods

### 4.1. Plant Material and Growth Conditions

*Arabidopsis thaliana* ecotype Columbia-0 (Col-0) was used as the wild type. A homozygous *TPPI* T-DNA insertion mutant (SAIL-354-D09), hereafter referred to as *tppi*, was obtained from the Nottingham Arabidopsis Stock Centre (NASC) and identified by PCR using gene-specific and T-DNA border primers. A schematic representation of the *TPPI* gene and the position of the T-DNA insertion are shown in Supplementary Figure S1a. Loss of *TPPI* transcript accumulation in the mutant was confirmed by RT-PCR using total RNA extracted with the RNeasy Plant Mini Kit (Qiagen, Chatsworth, CA, USA), followed by first-strand cDNA synthesis using SuperScript III Reverse Transcriptase (Invitrogen, Carlsbad, CA, USA). To rescue the *TPPI* knockout, the *tppi* mutant was transformed with a construct containing the *TPPI* CDS. For overexpression, the full-length *TPPI* coding sequence was cloned into the binary vector pBA002 under the control of the CaMV 35S promoter and transformed into Col-0 plants using the same *Agrobacterium tumefaciens*-mediated floral dip method. Seven independent *TPPI*-OEX lines were generated and screened for *TPPI* expression. All independent lines showed elevated *TPPI* transcript levels relative to WT and no obvious phenotypic differences from one another. Given this consistency, a representative homozygous *TPPI*-OEX line was selected for the detailed analyses presented in this study. *TPPI* transcript levels in the mutant, complemented line, and *TPPI-OEX* plants were verified by RT-PCR and are shown in Supplementary Figure S1b. The primer sequences used for genotyping, generation, and molecular characterization of the transgenic lines are provided in Supplementary Table S1.

Plants were grown in soil under controlled conditions at 22 °C. For flowering-time measurements and nitrogen-related experiments, plants were cultivated under long-day conditions (16 h light/8 h dark). For flowering-related gene-expression analysis, whole shoots were harvested from 2-week-old plants at ZT14 (14 h after lights-on), a time point selected to capture high CO/FT expression under long-day conditions. Plants used for chlorophyll-content and photosynthetic analyses were grown under the photoperiod specified for each experiment. For nitrogen-starvation experiments, plants were grown in soil for 3 weeks and then transferred to rock wool supplied with either complete Hoagland solution or nitrogen-free Hoagland solution prepared as previously described [36]. Plants were subjected to nitrogen starvation for 5 days for qRT-PCR analysis or 7 days for anthocyanin measurements. For qRT-PCR, nitrogen-starved plants were resupplied with complete Hoagland solution containing nitrate, and comparable rosette leaf tissue was harvested from each genotype 3 h after nitrate resupply. Unless otherwise specified, plant material collected for molecular and biochemical analyses was immediately frozen in liquid nitrogen and stored at −80 °C until analysis. Samples used for chlorophyll measurements were processed separately as described below.

### 4.2. qRT-PCR

Quantitative reverse transcription PCR (qRT-PCR) was performed as previously described [37]. Total RNA was isolated using the RNeasy^®^ Plant Mini Kit (Qiagen, Chatsworth, CA, USA), and cDNA was synthesised using the High-Capacity cDNA Reverse Transcription Kit (Applied Biosystems, Foster City, CA, USA). MicroRNA was isolated using the mirVana miRNA Isolation Kit (Invitrogen, Carlsbad, CA, USA) and cDNA was made using the TaqMan MicroRNA reverse transcription kit (Applied Biosystems). Real-time PCR reactions were assayed using a Light Cycler 96 Sequence Detection System (Roche Diagnostics, Mannheim, Germany). Each 25 µL reaction contained 12.5 µL TaqMan buffer (Applied Biosystems, includes ROX as a passive reference dye), 8.75 µL H_2_O, 2.5 µL cDNA and 1.25 µL primers. Predesigned TaqMan^®^ Gene Expression assays were used for the following *Arabidopsis thaliana* genes (Applied Biosystems assay identification numbers are given in parentheses): flowering-related genes: *FT* At1g65480 (At02224075), *FLC* At5g10140 (At02272498), *MYB33* At5g06100 (At02337117), *SOC1*/*AGL20* At2g45660 (At02263356), *CO* At5g15840 (At02200179), *SPL3* At2g33810 (At02204412), *MIR156A* At2g25095 (Assay ID000333). Nitrogen-related genes: *NIA1* At1g77760 (At02288113_g1), *NIA2* At1g37130 (At02194330_g1), *NRT1.1* At1g12110 (At02284072_m1), and *NRT2.1* At1g08090 (At02333192_g1). The following standard cycling conditions were used for amplification: 2 min at 50 °C, 10 min at 95 °C, followed by 40 cycles of 15 s at 95 °C and 1 min at 60 °C. The qPCR results were analysed using LightCycler 96 analysis software 1.1 (Roche). The comparative C_t_ method was used for relative quantification with ubiquitin At3g02540 (At02163241_g1) and *ACT8* At1g49240 (At02270958) as reference genes. Relative quantity (RQ = 2^−ΔΔCt^) of any gene was calculated relative to WT (calibrator). Expression levels are presented relative to WT, which was set to 1.

### 4.3. Nitrate Reductase Assay

Plants were grown in soil and supplied with complete Hoagland solution containing 15 mM total nitrate for 5 weeks under short-day conditions (8 h light/16 h dark). For NR activity measurements, plants were dark-adapted overnight and then exposed to light for 3 h before the first sample was harvested. Plants were subsequently transferred to darkness for 45 min and a second sample was collected, followed by 30 min of re-illumination before collection of the final sample. Rosette leaves (0.5 g fresh weight) were harvested at each sampling point, immediately frozen in liquid nitrogen, stored at −80 °C until analysis, and subsequently homogenised in a mortar with 1 mL of cold extraction buffer containing 100 mM HEPES-KOH, 1 mM EDTA, and 7 mM cysteine. The extract was filtered through Miracloth (Calbiochem, Merck, Darmstadt, Germany), and 50 μL of the filtrate was assayed in a total reaction volume of 700 μL containing 2 mM KNO_3_, 200 μM NADH and 2 mM EDTA to determine total NR activity (phosphorylated plus non-phosphorylated NR). To measure actual NR activity (non-phosphorylated NR), the assay was performed in the presence of 6 mM MgCl_2_. Reactions were incubated at 25 °C for 10 min. Nitrite formation was determined after the addition of 700 μL of 1% sulphanilamide and 0.02% N-(1-naphthyl)ethylenediamine dihydrochloride in 1.5 N HCl, and absorbance was measured at 540 nm using a SmartSpec 3000 spectrophotometer (Bio-Rad Laboratories, Hercules, CA, USA). An absorbance of 1.0 corresponds to 27 μmol NO_2_^−^ g^−1^ fresh weight h^−1^. The NR activation state was calculated as the ratio of actual activity (Mg^2+^ assay) to total NR activity (EDTA assay) multiplied by 100 [26].

### 4.4. Nitrate Measurement

Sample nitrate content was determined using the salicylic acid method. First, 100 mg of fresh Arabidopsis leaves was transferred to a test tube containing 10 mL of ultrapure water and placed in a boiling water bath for 30 min. Afterwards, the mixture was cooled to room temperature and filtered into a 25 mL volumetric flask, and the volume was adjusted to 25 mL with ultrapure water. Next, 0.1 mL of the filtered supernatant was transferred to a test tube, and 0.4 mL of 5% salicylic acid/sulfuric acid solution was added. Subsequently, 9.5 mL of 8% NaOH solution was added slowly, and the absorbance of the solution was measured at 410 nm [38].

### 4.5. Anthocyanin Measurement

Rosette leaf tissue weighing 0.050 g (fresh weight) was extracted overnight at 4 °C with shaking in 300 µL of 1% (*v*/*v*) hydrochloric acid in methanol. Following extraction, 200 µL of distilled water was added to the mixture and thoroughly mixed. Then, 500 µL of chloroform was added, mixed well, and the mixture was centrifuged at 15,000 *g* for 2 min. The upper layer (400 µL) was transferred to a new microtube, and 600 µL of 1% (*v*/*v*) hydrochloric acid in methanol was added and the sample was centrifuged again to remove particulates. The resulting supernatant was used for absorbance measurements at 530 nm and 657 nm. Relative anthocyanin concentrations were determined by subtracting the absorbance at 657 nm from the absorbance at 530 nm, using a method modified from [36].

### 4.6. Chlorophyll Content Measurement

Fresh leaves from Arabidopsis WT and mutant plants were harvested and transferred into individual test tubes. For pigment extraction, N,N-dimethylformamide (DMF) was added to each tube at 10 mL DMF per gram of fresh weight, and the samples were incubated overnight at 4 °C under constant shaking. Following extraction, the extract was diluted 1:10 with DMF and used for spectrophotometric analysis. The full absorption spectrum of the diluted extract was recorded using a UV-2401PC UV–Vis spectrophotometer (Shimadzu Corporation, Kyoto, Japan), scanning wavelengths from 400 to 700 nm. Chlorophyll *a* and *b* contents were determined from absorbance at 664.5 and 647 nm, respectively, according to Inskeep and Bloom [39], using the following equations:

Chlorophyll *a* (Chl *a*) = 12.70 × A_664.5_ − 2.79 × A_647_

Chlorophyll *b* (Chl *b*) = 20.70 × A_647_ − 4.62 × A_664.5_

Total chlorophyll (Chl *a* + *b*) = 17.90 × A_647_ + 8.08 × A_664.5_

where A_664.5_ and A_647_ are the absorbance values at 664.5 and 647 nm, respectively. Chlorophyll concentrations calculated from these equations were converted to mg g^−1^ fresh weight by accounting for the extraction volume, dilution factor, and tissue fresh weight.

### 4.7. Chlorophyll Fluorescence and P700 Measurements

Photosynthetic measurements were performed on leaves of 4-week-old Arabidopsis plants grown at a photon flux density of 100 µmol photons m^−2^ s^−1^. After dark adaptation overnight, PSI and PSII parameters were recorded simultaneously at room temperature for WT and mutant plants using a Dual-PAM-100 measuring system (Heinz Walz GmbH, Effeltrich, Germany). PSI photosynthetic parameters were measured following Klughammer and Schreiber, with P700+ signals ranging from a minimum (P700 fully reduced) to a maximum level (P700 fully oxidised). The maximum, Pm, was determined by applying a 200 ms saturation pulse at instrument intensity setting 6 after pre-illumination with far-red light for 10 s, and Pm′ was determined similarly under actinic light. PSI parameters included the quantum yield of PSI non-photochemical energy dissipation due to donor-side limitation, Y(ND) = P/Pm, and the quantum yield of non-photochemical energy dissipation due to acceptor-side limitation, Y(NA) = (Pm − Pm′)/Pm. For PSII measurements, chlorophyll fluorescence was recorded to determine the maximum quantum yield (Fv/Fm) after dark adaptation by measuring minimal fluorescence (Fo) and maximum fluorescence (Fm) using a 200 ms saturating pulse at instrument intensity setting 6. The effective quantum yield of PSII (ΦPSII) under light conditions was calculated as (Fm′ − Fs)/Fm′, where Fm′ is the maximum fluorescence in the light-adapted state and Fs is the steady-state fluorescence. For photosynthetic induction measurements, actinic light was set to 115 µmol photons m^−2^ s^−1^. Measurements were performed on 10 independent leaves per genotype. Data are presented as mean ± SE.

### 4.8. Statistical Analysis

Data are presented as means ± SE. Multi-group comparisons involving a single experimental factor were analysed by one-way ANOVA followed by Tukey’s HSD test for multiple comparisons. Factorial experiments were analysed by two-way ANOVA, with the factors specified for each experiment. Light-response curves and photosynthetic induction curves were analysed by two-way repeated-measures ANOVA, with genotype as the between-subject factor and PAR or time, respectively, as the within-subject factor. The number of biological replicates (n) is specified in each figure legend, and individual data points represent independent biological replicates (individual plants or leaves, as indicated). Technical replicates, where applicable, were averaged and were not treated as independent biological replicates. Statistical significance was accepted at *p* < 0.05.

## Data Availability

All data generated or analysed during this study are included in the article and its Supplementary Materials.

## Supplementary Materials

The following supporting information can be downloaded at https://www.mdpi.com/article/10.3390/plants15172559/s1. Figure S1. Molecular characterisation of *tppi* mutant, complemented line and *TPPI-OEX* plants. Figure S2. Anthocyanin accumulation in *TPPI-OEX* plants and basal nitrate reductase gene expression in *tppi* plants. Figure S3. Pale-leaf phenotype and photosynthetic induction kinetics in *tppi* plants. Figures S4–S8. Mathematical model of TPP crosstalk, analytical derivations, and numerical simulations. The model and numerical simulations are documented by the Python scripts FigureS7.py and FigureS8.py, which generate the results presented in Figures S7 and S8. Table S1. Primer sequences used for genotyping, transcript verification, and generation of *TPPI* transgenic lines.
