# Supplementary material for: Trehalose-6-Phosphate Phosphatase I (TPPI) Regulates Floral Transition, Nitrogen Responses, and Photosynthetic Performance in Arabidopsis
Source: Plants (Basel). 2026 Aug 23;15(17):2559. doi: 10.3390/plants15172559 (PMC13566947; doi:10.3390/plants15172559)
Supplement: Supplementary file 1 [file plants-15-02559-s001.zip › Supplementary_Materials.pdf]

Supplementary Materials

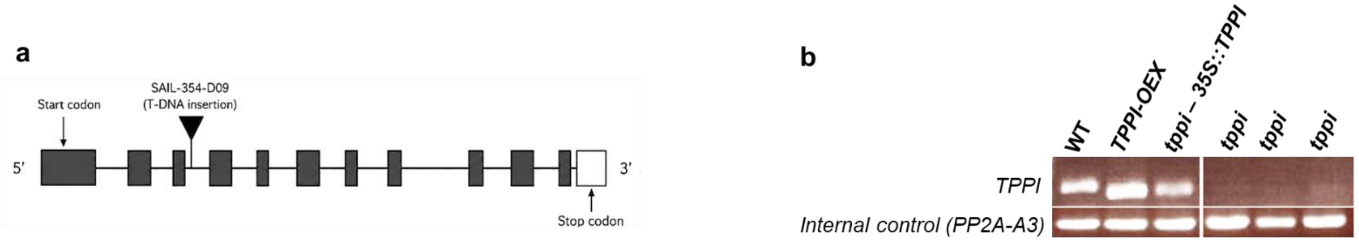

Figure S1. Molecular characterisation of the *tppi* mutant, complemented line and *TPPI-OEX* plants.
(a) Schematic representation of the *TPPI* gene structure showing the T-DNA insertion site in the *tppi* mutant. Exons are indicated as black boxes and introns as lines. (b) RT-PCR analysis of *TPPI* transcript levels in wild-type (WT), *tppi*, *tppi*+35S::*TPPI* (complemented) and *TPPI-OEX* plants.

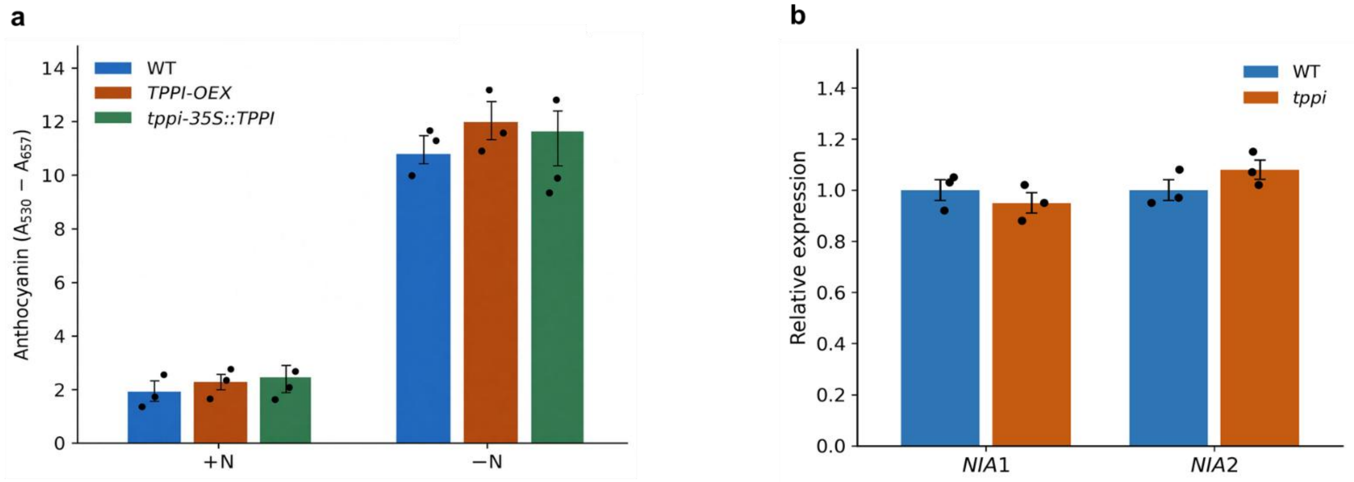

Figure S2. Anthocyanin accumulation in *TPPI-OEX* plants and complemented line and basal nitrate reductase gene expression in *tppi* plants. (a) Anthocyanin content ($A_{530} - A_{657}$) in rosette leaves of WT, the complemented line and *TPPI-OEX* plants under +N and −N conditions. Values are means ± SE (n = 3 biological replicates). Individual data points are shown. Data were analysed by two-way ANOVA. No significant genotype differences were detected. (b) Relative expression of *NIA1* and *NIA2* in WT and *tppi* plants under +N conditions. Expression was normalised to WT (= 1). Values are means ± SE (n = 3 biological replicates). Individual data points are shown. Data were analysed by one-way ANOVA. No significant differences were detected.

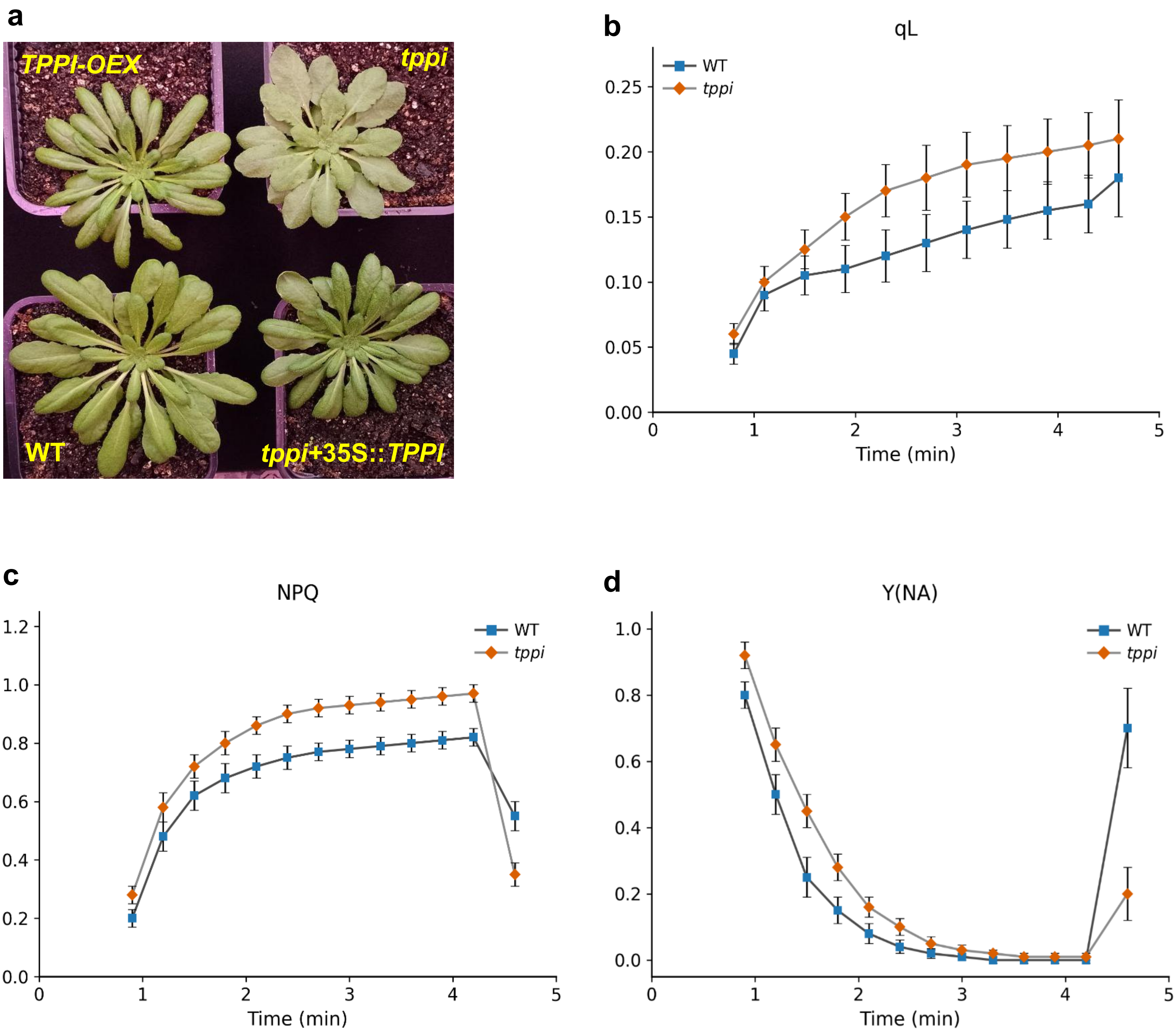

Figure S3. Pale-leaf phenotype and photosynthetic induction kinetics in *tppi* plants.
(a) Representative images of WT, *tppi*, the complemented line (*tppi*+35S::*TPPI*), and *TPPI-OEX* plants grown under short-day conditions, showing the pale-leaf phenotype of the *tppi* mutant. (b–d) Photosynthetic induction kinetics of (b) qL, (c) non-photochemical quenching (NPQ), and (d) PSI acceptor-side limitation [Y(NA)] in WT and *tppi* plants. Values are means ± SE (n = 10 independent leaves per genotype). Curves were analysed by two-way repeated-measures ANOVA, with genotype as the between-subject factor and time as the within-subject factor. Significant overall genotype effects were detected for qL (P = 0.00046), NPQ (P < 0.0001), and Y(NA) (P = 0.0213).

Modeling TPP crosstalk

All ten TPP isoenzymes are found to be enzymatically active and have redundant and non-reduntant roles [1]. As an illustration, the model we consider here (Fig S4) contains two redundant TPP isoenzymes described as 'TPP1' and 'TPP2'. However, we not only assume that they are only enzymatically active, but that they influence each other: that TPP1 is able to inhibit or activate (directly or indirectly) TPP2 by a factor f_1 and that TPP2 inhibits or activates TPP1 by factor f_2.

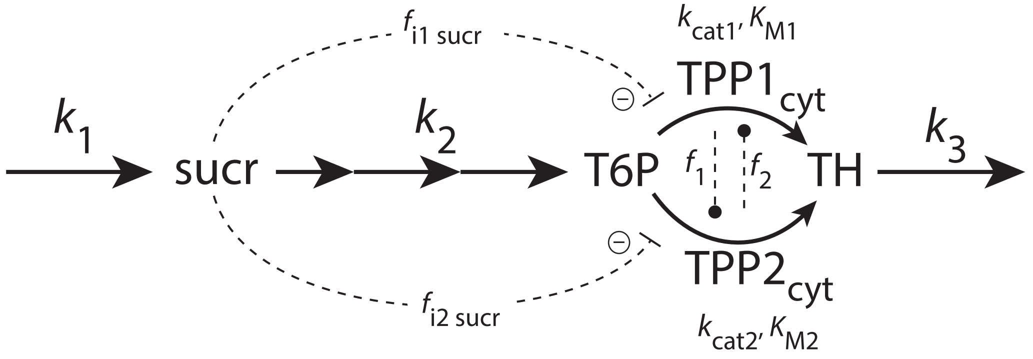

Figure S4: The model. Both TPP1 and TPP2 catalyze the conversion of trehalose-6-Phosphate (T6P) to trehalose (TH). The inhibition of the TPPs by sucrose [2] is included with inhibition factors $f_{i1\,sucr}$ and $f_{i2\,sucr}$. The dashed lines between TPP1 and TPP2 with solid bullet at the end indicate activations or inhibitions with factors f_1 and f_2. For rate equations, see text.

The rate equations are:

$$\frac{d\,(sucr)}{d\,t} = k_1 - k_2 \cdot (sucr) \tag{S1}$$

$$\frac{d\,(T6P)}{d\,t} = k_2 \cdot (sucr) - v_{TPP1} - v_{TPP2} \tag{S2}$$

$$\frac{d\,(TH)}{d\,t} = v_{TPP1} + v_{TPP2} - k_3 \cdot (TH) \tag{S3}$$

Note that the steady state of TH is given by the ratio k_1/k_3 and is independent of the rates how sucrose and T6P are turned over. This can be seen

by setting Eqs 1-3 to zero, sum them up, and solving for $(TH)_{ss}$.

The rate expressions for v_{TPP1} and v_{TPP2} are:

$$v_{TPP1} = \left(\frac{\overbrace{k_{cat1}\cdot(TPP1)}^{V_{max1}}\cdot(T6P)}{K_{M1}+(T6P)} \right)\cdot f_{i1\,sucr}\cdot f_2 = \left(\frac{V^*_{max1}\cdot(T6P)}{K_{M1}+(T6P)} \right)\cdot f_2 \qquad \text{(S4)}$$

$$v_{TPP2} = \left(\frac{\overbrace{k_{cat2}\cdot(TPP2)}^{V_{max2}}\cdot(T6P)}{K_{M2}+(T6P)} \right)\cdot f_{i2\,sucr}\cdot f_1 = \left(\frac{V^*_{max2}\cdot(T6P)}{K_{M2}+(T6P)} \right)\cdot f_1 \qquad \text{(S5)}$$

with $V^*_{max1}=V_{max1}\cdot f_{i1\,sucr}$, $V^*_{max2}=V_{max2}\cdot f_{i2\,sucr}$, and $f_{i1\,sucr}=K^{sucr}_{i1}/(K^{sucr}_{i1}+(sucr))$, $f_{i2\,sucr}=K^{sucr}_{i2}/(K^{sucr}_{i2}+(sucr))$. The concentrations of TPP1 and TPP2 are considered to be constant. K^{sucr}_{i1} and K^{sucr}_{i2} are inhibition constants.

The factors f_1 and f_2 are either inhibition or activation factors. In case they are inhibition factors they are described in a generic form as mixed TPP1 or TPP2 inhibitors with inhibition constants K_i [3], i.e.

$$f_i = f^{inhib}_{1\,or\,2} = \frac{K_i}{K_i+TPP(1\,or\,2)} \qquad \text{(S6)}$$

When f_1 or f_2 act as activators we describe them in form of a mixed activation where both activation constants have the value (K_a) [3], i.e.

$$f_a = f^{act}_{1\,or\,2} = \frac{TPP(1\,or\,2)}{K_a+TPP(1\,or\,2)} \qquad \text{(S7)}$$

Fig S5 illustrates how f_i and f_a depend on their respective activation and inhibition constants K_i or K_a when the TPP's are constant (=1.0).

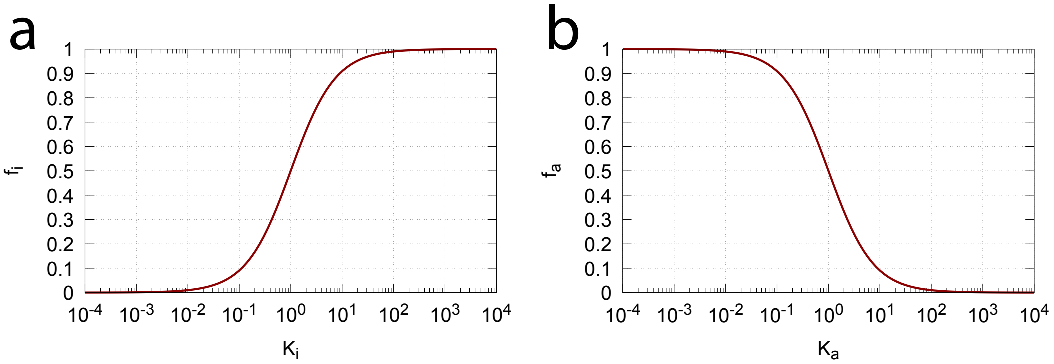

Figure S5: Dependence of f_i (panel a) and f_a (panel b) as a function of their respective inhibition and activation constants, K_i (Eq s6) and K_a (Eq s7). The concentration of TPP is 1.0.

First-order kinetics with respect to T6P

To make a first 'on back of an envelope' calculation how a TPP knockout affects the steady state of T6P, we make the following assumptions:

(A) The TPPs show first-order kinetics with respect to their substrate T6P. This means that substrate concentrations are low in comparison with Michaelis constants.

(B) Both TPPs have the same kinetic properties, i.e. $V^*_{max1}=V^*_{max2}=V^*_{max}$ and $K_{M1}=K_{M2}=K_M$

In case both TPP1 and TPP2 are enzymatically active the rate equation of T6P becomes:

$$\frac{d\,(T6P)}{d\,t} = k_2\cdot(sucr) - (T6P)\left(\frac{V^*_{max1}}{K_{M1}}\cdot f_2 + \frac{V^*_{max2}}{K_{M2}}\cdot f_1\right) \tag{S8}$$

$$= k_2\cdot(sucr) - (T6P)\cdot\frac{V^*_{max}}{K_M}\,(f_1 + f_2) \tag{S9}$$

The T6P steady state value is in this case:

$$(T6P)_{ss} = \frac{k_2\cdot(sucr)}{\frac{V^*_{max}}{K_M}\cdot(f_1 + f_2)} \tag{S10}$$

In case TPP1 is knocked out its V^*_{max} values disappears, but also does the activation/inhibition factor f_1 by TPP1. The T6P steady state now becomes:

$$(T6P)_{ss,\,ko} = \frac{k_2{\cdot}(sucr)}{\frac{V^*_{max}}{K_M}} \tag{S11}$$

We introduce α, which is the ratio between $(TH)_{ss}$ (Eq s10) and $(T6P)_{ss}$ $_{ko}$, i.e. using Eqs 10 and 11 gives:

$$\alpha = \frac{(T6P)_{ss}}{(T6P)_{ss,\,ko}} = \frac{1}{f_1{+}f_2} \tag{S12}$$

With respect to α we can distinguish two scenarios:

(i) $\alpha \geq 1$: $(T6P)_{ss,\,ko} \leq (T6P)_{ss}$

(ii) $1 < \alpha \leq \frac{1}{2}$: $(T6P)_{ss,\,ko} > (T6P)_{ss}$

Note, when α is $\frac{1}{2}$ in case (ii), we have that f_1=1 and f_2=1. In this case there is no inhibition, as K_i is negligible and the inhibitor TPP does not bind to the catalyzing TPP. In case of mutual activation, f_1=1 and f_2=1 means that TPP1 and TPP2 are both fully activated.

Michaelis-Menten kinetics

If we turn to Michaelis-Menten kinetics, but keep above assumption (B) that the TPPs have the same V_{max} and K_M values, we get from Eqs 2, 4, and 5:

$$\frac{d\,(T6P)}{d\,t} = k_2{\cdot}(sucr) - \frac{V^*_{max}{\cdot}(T6P)}{K_M{+}(T6P)}\,(f_1 + f_2) \tag{S13}$$

At steady state we can introduce γ as

$$\gamma = \frac{(T6P)_{ss}}{K_M{+}(T6P)_{ss}} = \frac{k_2{\cdot}(sucr)}{V^*_{max}(f_1{+}f_2)} \tag{S14}$$

and solve $(T6P)_{ss}$ in terms of γ, i.e.:

$$(T6P)_{ss} = K_M\left(\frac{\gamma}{1{-}\gamma}\right) \tag{S15}$$

When TPP1 is knocked out the rate equation for T6P becomes:

$$\frac{d\,(T6P)}{d\,t} = k_2{\cdot}(sucr) - \frac{V^*_{max}{\cdot}(T6P)}{K_M{+}(T6P)} \tag{S16}$$

and the factor $(f_1{+}f_2)$ disappears. At steady state, we can now introduce δ as:

$$\delta = \frac{(T6P)_{ss,\,ko}}{K_M{+}(T6P)_{ss,\,ko}} = \frac{k_2{\cdot}(sucr)}{V^*_{max}} \tag{S17}$$

with

$$(T6P)_{ss,\,ko} = K_M\left(\frac{\delta}{1{-}\delta}\right) \tag{S18}$$

Calculating now α, gives:

$$\alpha = \frac{(T6P)_{ss}}{(T6P)_{ss,\,ko}} = \left(\frac{\gamma}{\delta}\right)\cdot\left(\frac{1{-}\delta}{1{-}\gamma}\right) = \frac{V^*_{max}{-}k_2{\cdot}(sucr)}{V^*_{max}(f_1{+}f_2){-}k_2{\cdot}(sucr)} \tag{S19}$$

Considering Eq 19 we can distinguish again two scenarios:

(i) $f_1{+}f_2{<}1 \;\Rightarrow\; \alpha{>}1$ & $(T6P)_{ss,\,ko}{<}(T6P)_{ss}$

(ii) $f_1{+}f_2{\geq}1 \;\Rightarrow\; \alpha{\leq}1$ & $(T6P)_{ss,\,ko}{\geq}(T6P)_{ss}$

Fig S6 shows how α (red line) depends on $(f_1{+}f_2)$ when V_{max}=100.0, K^{sucr}_{i1}=K^{sucr}_{i2}=0.1, k_2=1.0, and $(sucr)$=1.0.

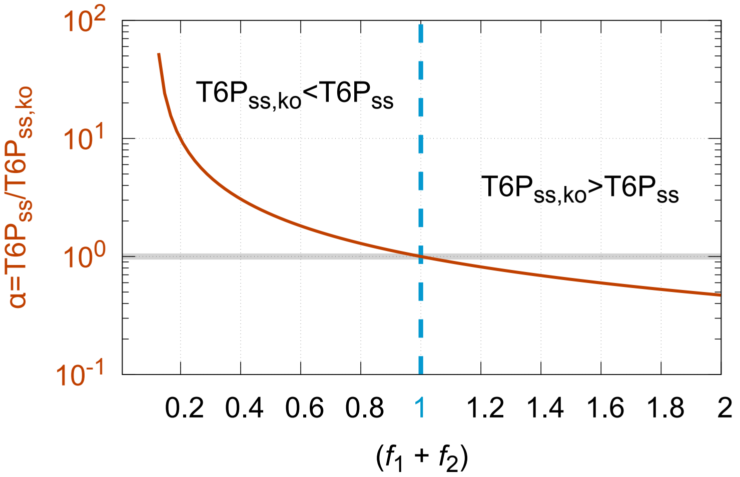

Figure S6: α as a function of $(f_1{+}f_2)$ as described by Eq s19.

Python scripts

When the above assumption (B) is relaxed and all rate parameters may have different values the calculation of T6P steady states become more intricate involving quadratic equations with respect to T6P. Instead, Eqs 1-3 can be solved numerically. The Python script `FigS7.py` (included in the Supporting Material) solves the rate equations and plots the T6P concentration against time. Fig S7 shows the results of such a calculation. In phase 1 both TPP1 and TPP2 are present and inhibit each other. In phase 2 TPP1 is set to zero to mimic a TPP1 knock out. While trehalose levels (TH) regain their original value, trehalose-6-phosphate levels (T6P) are decreased since the sum of f_1 and f_2 (now being inhibition factors) are lower than one, as indicated by Fig S6. In phase 3, TPP1 receives its original value and T6H settles to its original value. To run the script, see instructions inside the file.

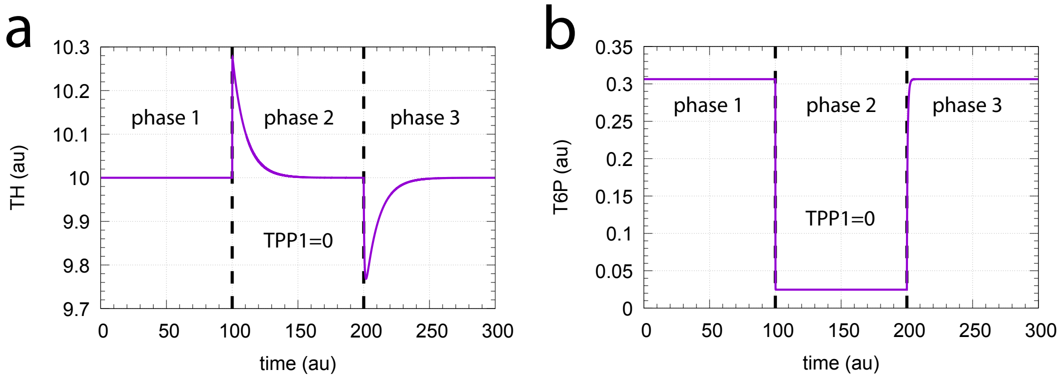

Figure S7:Results by solving the rate equations s1-s3 numerically. Rate constants, phase 1: k_1=k_2=1.0, k_3= 0.1, k_{cat1}=k_{cat2}=100.0, K_{M1}=K_{M2}=0.2, K_{i1}^{sucr}=K_{i2}^{sucr}=0.1, K_i^{TPP1}=0.1 (TPP1 inhibition constant acting on TPP2 leading to f_1), K_i^{TPP2}=0.1 (TPP2 inhibition constant acting on TPP1 leading to f_2), TPP1=TPP2=1.0. Initial concentrations: $sucr_0$=1.0, $T6P_0$=0.3063, TH_0=10.0. At the beginning of phase 2 (time=100.0) TPP1 is set to zero while all other rate parameters are unchanged. Now $T6P_{ss}$ goes to lower values compared with phase 1, while TH_{ss} reaches its original value from phase 1. In phase 3 starting at time=200.0 TPP1 is set again to 1.0 and steady states settle to the values of phase 1.

Fig S8 shows the results when there is no inhibition between TPP1 and

TPP2. In this case the inhibition factors f_1=f_2=1.0, which is achieved by setting K_i^{TPP1}=K_i^{TPP2}=1.0×10^6 (see Fig S5a). Now, when TPP1 is knocked out, the T6P steady state concentration in phase 2 increases, because the total flux from T6P to TH is reduced.

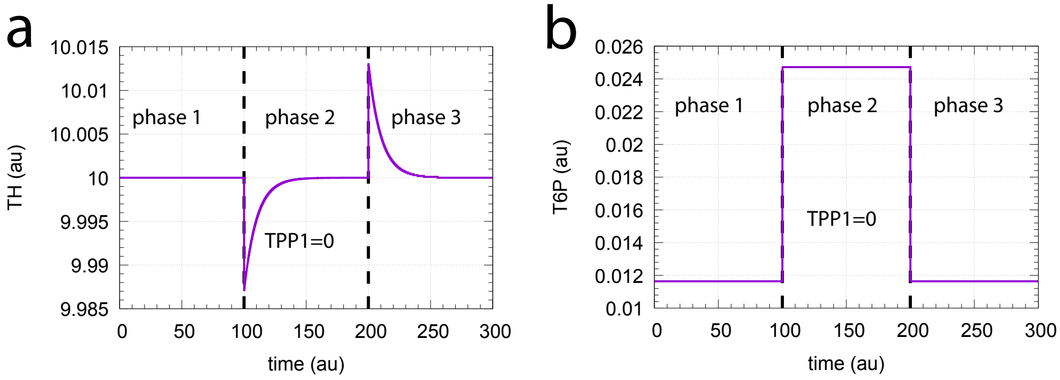

Figure S8: Same conditions as in Fig S7 with the exception that K_i^{TPP1}=K_i^{TPP2}=1.0×10^6, which abolishes the inhibitions between TPP1 and TPP2. When TPP1 is knocked out in phase 2 the steady state of T6P increases. Initial concentrations: $sucr_0$=1.0, $T6P_0$=1.1640×10^{-2}, TH_0=10.0. Run `FigS8.py` to get these results.

Bibliography

[1] Hilde Van Houtte, Lorena López-Galvis, Lies Vandesteene, Tom Beeckman, and Patrick Van Dijck. Redundant and non-redundant roles of the trehalose-6-phosphate phosphatases in leaf growth, root hair specification and energy-responses in arabidopsis. *Plant Signaling & Behavior*, 8(3):e23209, 2013.

[2] Yangzhi Liu, Boqiang Li, Tong Chen, Shiping Tian, and Zhanquan Zhang. The synthesis, degradation and biological function of trehalose-6-phosphate. *Stress Biology*, 5(1):38, 2025.

[3] A. Cornish-Bowden. *Fundamentals of Enzyme Kinetics. Fourth Edition.* Portland Press, London, 2012.

Table S1. Primer sequences used for genotyping, transcript verification, and generation of *TPPI* transgenic lines.

Purpose	Primer name	Sequence (5′–3′)
tppi (SAIL-354-D09) genotyping	SAIL_354_D09-LP	TTCAATCATTGGACGGATTTC
tppi (SAIL-354-D09) genotyping	SAIL_354_D09-RP	ACGACAGATGCAACATCCTTC
T-DNA border genotyping	SAIL LB-1	TAGCATCTGAATTTCATAACCAATCTCGATACAC
TPPI transcript verification by RT-PCR	TPPI-RT-F	TCATGTCAAGCAAGATGAGAAGAACAGT
TPPI transcript verification by RT-PCR	TPPI-RT-R	TCGACGCAGCGAAAGTGCAC
Full-length *TPPI* cloning for complementation/OEX	TPPI-F	AATTAATTAACATGTCAGCTAGTCAAAACATTGTC
Full-length *TPPI* cloning for complementation/OEX	TPPI-R	AAACTAGTTCACATTCTTGGCTGCATTTGTTT
